# Mitochondrial Photobiomodulation as a Neurotherapeutic Strategy for Epilepsy

**DOI:** 10.3389/fneur.2022.873496

**Published:** 2022-06-16

**Authors:** Fabrízio dos Santos Cardoso, Francisco Gonzalez-Lima, Norberto Cysne Coimbra

**Affiliations:** ^1^Laboratory of Neuroanatomy and Neuropsychobiology, Department of Pharmacology, Ribeirão Preto Medical School of the University of São Paulo, Ribeirão Preto, Brazil; ^2^Department of Psychology and Institute for Neuroscience, The University of Texas at Austin, Austin, TX, United States

**Keywords:** photobiomodulation, low-level laser therapy, epilepsy, seizure, mitochodria, oxidative stress

## Introduction

Discovering that photobiomodulation (PBM) is neuroprotective and has augmentation effects on human neurocognitive functions has been groundbreaking (1). Transcranial PBM with near-infrared light at low irradiance (mW/cm^2^) and high energy density or fluence (J/cm^2^) modulates neural functions in a non-thermal way that may have therapeutic effects on various neurological disorders (2). Epilepsy is a brain disorder characterized by a persistent predisposition to generate epileptic seizures and by neurological, cognitive, and psychosocial consequences (3), in addition to postictal antinociception (4–7) and psychiatric comorbidities (8, 9). It is the fourth most common neurological condition in the world. An estimated 70 million people are suffering from some type of epileptic syndrome (10–12). We propose that transcranial PBM may be developed as a new non-invasive therapeutic strategy for epilepsy based on the following: (1) its well-documented mitochondrial mechanism of action relevant to epilepsy, (2) its beneficial neurocognitive effects in humans, and (3) the promising findings from two recent PBM studies in different epilepsy models.

## Mitochondrial Dysfunction in Epilepsy

One hypothesis to explain the role of mitochondria in epilepsy is linked to metabolic and energy changes after acute seizures and during chronic epilepsy (13–19). For example, Mueller et al. (14) noted that redox status measured by reduced and oxidized forms of glutathione changes to a more oxidized state in the brain and plasma of epileptic patients. During seizure activity, an acute increase in glucose metabolism and cerebral blood flow is observed in patients with temporal lobe epilepsy (TLE) (17), the most prevalent form of acquired epilepsies (20). In addition, in the study conducted by Vielhaber et al. (19), it was noted that the hypometabolism observed in patients with epilepsy is associated with low levels of mitochondrial N-acetyl aspartate in the CA3 hippocampal subfield. Reduced levels of NAD(P)H were also observed in CA1, CA2, and the subiculum of patients with TLE (21).

Studies performed in laboratory animals have suggested mitochondrial dysfunction and oxidative stress as a key mechanism that follows seizures and contributes to epileptogenesis (20, 22, 23). After seizures there are many changes related to mitochondrial dysfunction and oxidative stress, including an acute increase in mitochondrial oxidative stress, excessive reactive oxygen species (ROS) production, increased oxidation of cellular macromolecules, mitochondrial DNA damage, decreased activity of the electron transport chain (ETC), and increased nitric oxide (NO) generation in the cerebral cortex (24) and hippocampus (22, 25–27). Also, studies have shown a decrease in hippocampal ETC complex I and IV activity and oxidative stress in CA1 and CA3 during chronic epilepsy (15, 16, 18).

In view of this, targeting mitochondrial dysfunction and oxidative stress with PBM may provide a new therapeutic strategy to attenuate seizure activity, impairments linked to neuronal loss, and cognitive function (30).

## Mitochondrial Mechanism of Action of Photobiomodulation

Generally, PBM, also known as low-level laser therapy (31), is a non-invasive method that has been shown to modulate neuronal functions, including mitochondrial energy metabolism, proliferation, differentiation, and apoptosis (32, 33). The mechanism of action of PBM primarily involves a photonic biochemical effect on mitochondrial respiration and oxidative stress (34). The major acceptor of red-to-near-infrared photons inside cells is the mitochondrial enzyme cytochrome c oxidase (CCO, also called ETC complex IV), which is considered a fundamental molecule for the action of PBM (35–38).

Photonic oxidation of CCO by transcranial PBM with a near-infrared laser has been demonstrated *in vivo* in the human brain (39, 40). PBM can induce a series of beneficial cellular events, such as the increase in oxidative phosphorylation for ATP production, increased permeability of the mitochondrial membrane, a brief increase in ROS, and activation of mitochondrial signaling pathways linked to neuroprotection and cell survival (2, 41). In addition, NO released by CCO is able to stimulate ATP production by increasing mitochondrial membrane potential and oxygen consumption (35, 36, 38, 42–44), as well as triggering a physiological hemodynamic response to increasing delivery of oxygen to the human brain (39, 40). However, mechanisms other than CCO may mediate PBM effects under certain conditions, as suggested by the extensive metabolomic effects of PBM on the rat brain (45).

## Neurocognitive Effects of Photobiomodulation in Humans

Many human studies have demonstrated the potential of transcranial PBM for the augmentation of neurocognitive functions under several conditions (1, 46–54). Studies using laboratory animals have also documented interesting results of brain PBM (45, 55, 56). For example, our research group submitted aged rats to PBM with transcranial laser for 58 consecutive days and we noted that laser treatment was able to rejuvenate the spatial mnemonic damage of the aged rats and modulate brain levels of inflammatory markers (56). In addition, this same laser treatment protocol increased the brain metabolic pathways of young rats and restored the brain metabolic pathways of aged rats to the levels of younger rats (45).

## Studies of Photobiomodulation in Epilepsy Models

Regarding epilepsy, there have been two recent pre-clinical studies showing beneficial effects of PBM in different epilepsy models (Table 1).

**Table 1:** Recent evidence of PBM benefits in epilepsy models.

**Reference**	**Epilepsy model**	**Light source**	**Treatment/Parameters**	**Outcomes**
Tsai et al. (28)	Sprague-Dawley rats, Pentylenetetrazole-induced epilepsy	GaAlAs diode laser	Center wavelength: 808 nm Operating mode: continuous Average radiant power: 110 mW Irradiance at aperture: 1.333 W/cm^2^ Beam spot size: 0.0825 cm^2^ Exposure duration: 100 s Radiant exposure: 133.3 J/cm^2^ Number of sessions: 1 Total radiant energy: 11 J	Photobiomodulation attenuated the mean seizure score and reduced the incidence of status epilepticus and mortality.
Vogel et al. (29)	Wistar rats, Photothrombotic stroke-induced epilepsy	LED	Center wavelength:780 nm Average radiant power: 10 mW Irradiance at aperture: 0.083 W/cm^2^ Beam spot size: 0.12 cm^2^ Exposure duration: 120 s Radiant exposure: 10 J/cm^2^ Number of sessions: 24 Total radiant energy: 1.20 J	Photobiomodulation reduced both electrographic seizure duration and spikes number in the ipsilateral and contralateral cortices and ventral posteromedial thalamic nucleus.

First, Tsai et al. (28) noted that transcranial PBM at wavelength 808 nm was able to attenuate pentylenetetrazole-induced status epilepticus in peripubertal rats. In addition, PBM reduced the apoptotic ratio of parvalbumin-labeled interneurons and alleviated the aberrant extent of parvalbumin-labeled unstained somata of principal cells in the hippocampus. Second, Vogel et al. (29) observed that PBM reduces epileptiform discharges after a stroke (29). They showed that a 780 nm wavelength laser treatment for 2 months after induction of photothrombotic stroke reduced late epileptic electrographic seizures, as well as the number of spikes in the ipsilateral and contralateral cortices and in the ventral posteromedial thalamic nuclei. Although there is a possibility that PBM could trigger epileptic seizures, there is no evidence in support for this, and the two studies evaluating PBM effects on seizure models have found that PBM reduces seizures.

Although these studies present interesting behavioral findings on PBM in epilepsy (28, 29), evidence regarding mitochondrial functions is still lacking. This line of reasoning would be interesting since studies show that mitochondrial damage under various conditions is restored by PBM. Furthermore, this restoration is accompanied by an improvement in behavioral performance (57–59). In fact, PBM increases mitochondrial membrane potential, contributing to an increase in ATP production and a brief increase in ROS (34, 60, 61). In addition, ROS and other mediators of PBM, such as NO and cyclic adenosine monophosphate (cAMP), activate transcription factors. In this sense, after PBM, CCO stimulates ATP synthesis (62, 63). Extracellular ATP is also a neurotransmitter (64) that participates in many signaling pathways, known as purinergic signaling (65). NO acts by stimulation of guanylate cyclase to form cyclic-GMP (cGMP), which induces Ca^++^ reuptake and the opening of calcium-activated potassium channels *via* protein kinase G (66). ROS is a mediator that at low concentrations and brief exposures is beneficial, and at high concentrations and long exposure, periods are harmful (67). When induced by PBM, ROS activates nuclear factor kappa B (NF-kB), which contributes to the increase in gene transcription, and consequently cellular processes, such as proliferation, migration, and cell death (60). cAMP can down-regulate the LPS-induced TNF-α synthesis at the transcriptional level (68–70). Also, cAMP exerts its cellular effects through the signaling of protein kinase A (PKA), cyclic nucleotide-gated channels (CNGC), and exchange proteins directly activated by cAMP (Epac) (71–73). Together, the upregulation of mitochondrial respiration that triggers these metabolic signaling cascades suggests that the long-term effects of PBM might be beneficial to treat the mitochondrial deficits found in epilepsy.

Although these results are promising, much more evidence of the effects of PBM on the epileptic brain is needed. When this evidence becomes available, then PBM may be translated to the clinic, but the evidence is too limited at this time.

## Conclusion

Transcranial PBM may treat the mitochondrial dysfunction in epilepsy by upregulating CCO, which is the terminal enzyme in mitochondrial respiration. This mitochondrial mechanism of action of PBM might benefit epilepsy because transcranial PBM is neuroprotective and improves human neurocognitive functions affected by epilepsy. This fascinating new intervention is safe and non-invasive and should be tested further to confirm if augmenting neuronal mitochondrial respiration is a neurotherapeutic strategy for epilepsy.

## Author Contributions

All authors listed have made a substantial, direct, and intellectual contribution to the work and approved it for publication.

## Funding

This work was supported by the Fundação de Amparo à Pesquisa do Estado de São Paulo (FAPESP) (grants 1996/8574-9 and 2009/00668-6), Conselho Nacional de Pesquisa e Desenvolvimento Tecnológico (CNPq) (grant 474425/2008-8) and Coordenação de Aperfeiçoamento de Pessoal de Ensino Superior (CAPES) (AUX-PE-PNPD 2400/2009; grant 23038.027801/2009-37). None of these organizations had a role in the study design, the collection, analysis and interpretation of the data, the writing of the report, or the decision to submit the paper for publication. FC was financially supported by FAPESP (Postdoctoral fellowship grant 2021/06473-4). NC Coimbra is a researcher from CNPq (PQ1A-level grants 301905/2010-0 and 301341/2015-0; PQ2-level grant 302605/2021-5). FG-L was supported by the Oskar Fischer Project Fund.

## Conflict of Interest

The authors declare that the research was conducted in the absence of any commercial or financial relationships that could be construed as a potential conflict of interest.

## Publisher's Note

All claims expressed in this article are solely those of the authors and do not necessarily represent those of their affiliated organizations, or those of the publisher, the editors and the reviewers. Any product that may be evaluated in this article, or claim that may be made by its manufacturer, is not guaranteed or endorsed by the publisher.
